# Clinical Features of Aortitis with Gastrointestinal Involvement

**DOI:** 10.31083/j.rcm2305162

**Published:** 2022-04-28

**Authors:** Mansour Altuwaijri, Abdulmajeed Altoijry

**Affiliations:** ^1^Division of Gastroenterology, Department of Medicine, College of Medicine, King Khalid University Hospital, King Saud University, 11451 Riyadh, Saudi Arabia; ^2^Division of Vascular Surgery, Department of Surgery, College of Medicine, King Saud University, 11472 Riyadh, Saudi Arabia

**Keywords:** vasculitis, aortitis, gastrointestinal manifestations

## Abstract

Few vascultides have a predilection for the aorta. Among those are Takayasu arteritis, Behcet’s disease, giant cell arteritis, and infectious aortitis. Diagnosis of aortitis requires a high index of suspicion since clinical features are atypical and nonspecific. However, many patients present with gastrointestinal manifestations owing to mesenteric involvement, intestinal infarction, and hepatitis. The most common vascultides that involve the aorta are Takayasu arteritis, Behcet’s disease, giant cell arteritis, and infectious arteritis. Herewith, we review the literature on epidemiology, gastrointestinal manifestations, and management of each form of aortitis that affects the gastrointestinal tract.

## 1. Introduction

Vasculitides is a group of diseases that present with inflammation of blood vessel walls and earn their classifications based on the size and type of the vessels involved, influencing the type and area of the ischemic injury [1].

Aortitis is vasculitis of the aortic wall and it can be a feature of systemic rheumatological, infectious or neoplastic disorders, and it can also be idiopathic [2]. Diagnosis of aortitis requires a high index of suspicion since clinical features are atypical and nonspecific [3]. While histopathology is the gold standard for diagnosing aortitis, tissue biopsy is not usually feasible, and correlating clinical findings with imaging and laboratory tests helps with the final diagnosis. As the clinical manifestation is nonspecific, aortitis could be easily overlooked if not suspected as part of the initial differential diagnosis.

In cases of aortitis presenting with gastrointestinal involvement, the large-vessel vasculitis could lead to widespread intestinal infarction and even involve other organs. Aortitis has a variable presentation ranging from mild abdominal pain to more severe and life-threatening bowel perforation and peritonitis. These manifestations could happen during diagnosis or could present later at a relapse time and are often isolated. The Five Factor Score (FFS) of 1996 described gastrointestinal manifestations as a major predictor of mortality in microscopic polyangiitis, polyarteritis nodosa and eosinophilic granulomatosis with polyangiitis (EGPA) with involvement of the central nervous system, kidneys and the heart [4]. Despite advances in diagnostics and management of aortitis, gastrointestinal manifestations remain, till this day, a serious problem.

Gastrointestinal manifestations are rarely the predominating features of systemic vasculitides but can rapidly become life-threatening. Aortitis with small vessel involvement can cause various gastrointestinal manifestations, including mucosal purpura (risk of hemorrhage), patchy granulomatous or ischemic ulcerations that can mimic inflammatory bowel disease (IBD) and can cause intestinal perforation. The most common vascultides that involve the aorta are Takayasu arteritis, Behcet’s disease, giant cell arteritis, and infectious arteritis.

## 2. Methods

We systematically searched MEDLINE (from 1940) and EMBASE (from 1972) up to the end of December 2021 using a comprehensive search strategy that combined MeSH terms and free text for “Aortitis”, “Gastrointestinal”, and “Takayasu”, “Behcet’s disease”, “giant cell arteritis”, “infectious”, and “mycotic”. Reference lists of all relevant studies, reviews, and letters were also searched to identify additional studies. The searches were limited to humans and adults.

Our inclusion criteria were broad and included prior systematic reviews and meta-analysis, clinical trials, cohort studies, case series, and case reports.

**Fig. 1.**
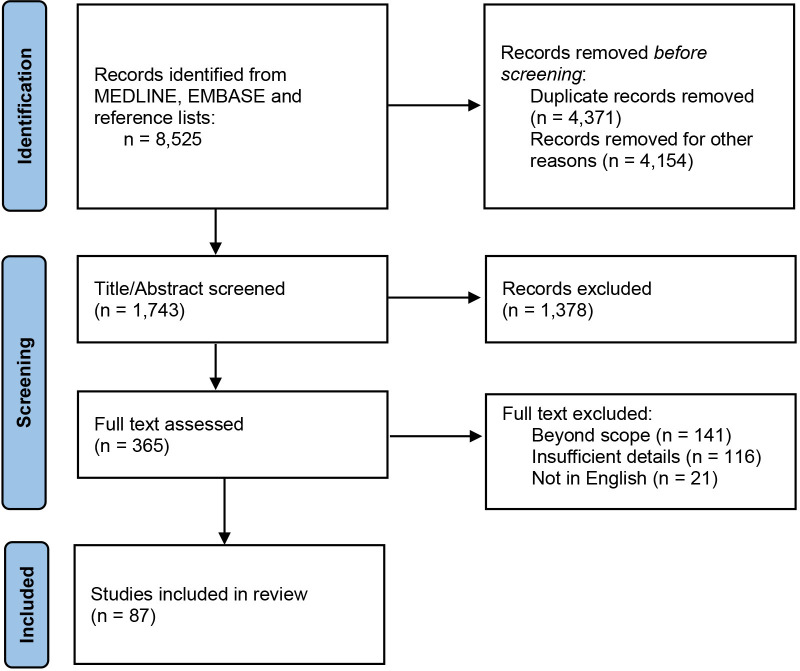
**PRIMSA flow diagram for clinical features of aortitis with gastrointestinal involvement**.

Both authors independently screened all titles and abstracts to identify potentially relevant articles. Disagreements were resolved by repeated review and discussion. They independently extracted data from the full-text articles using structured review forms that included epidemiology, diagnosis, gastrointestinal manifestations, and management. Articles that did not fulfill any of the review form items were excluded (Fig. 1).

## 3. Takayasu Arteritis

### 3.1 Epidemiology

The most common non-infectious aortitis (NIA) is takayasu arteritis (TKA). This is a rare obliterative and necrotizing idiopathic large vessel, segmental panarteritis [3]. It is most commonly found in women between the ages of 20 and 40 years in Southeast Asia, India and Mexico with Japan holding the highest prevalence [5, 6]. Due to its rarity, epidemiological data for incidence rates of TKA are limited. However, recent studies have put the incidence rate at 1–2 per million in Japan and 2.2 per million Kuwait [7, 8]. Recent European studies have put their specific incident rates between 0.4 and 1.3 per million, recognizing an increase in the recent years compared to older estimates from the European countries [9, 10, 11, 12, 13, 14]. Although its etiology is unknown, the frequency in specific populations and familial aggregation of TKA and its association with HLA alleles suggest involvement of genetic factors in the etiopathogenesis of TKA [15].

### 3.2 Diagnosis

Usually, TKA has a sabacute course lasting months to years. During this period, vascular involvement may progress and become symptomatic. In patients with TKA, constitutional symptoms such as weight loss, low grade fever and fatigability are common especially in the early period. Additionally, arthralgias and myalgias are occur in about one-half of cases. Tenderness of the carotid artery is also observed in 10–30% patients at presentation [16]. Peripheral pulses may be weak or absent, especially at the level of the radial arteries [17]. Ischemic ulceration and gangrene of the extremities my occur, but this is rare due to the fact that these complications are preceded by formation of collateral vessels. In all cases, limb claudication is common and involvement of the subclavian artery may be associated with subclavian steal syndrome, which gives rise to neurological symptoms and syncope during exercise [18].

Arterial stenoses manifests with a bruit which is usually audible over the subclavian, brachial, and carotid arteries. Stenosis also manifests with discrepancies in limb blood pressure of 10 mmHg or more. Therefore, patients with suspected TKA should have their blood pressure measured in all four limbs.

When stenosis involves coronary vessels, the most common feature is angina. Aortitis and coronary arteritis have been described in patients with TKA. In such cases, Myocardial infarction and death may occur.

Laboratory findings in TKA are nonspecific. Erythrocyte sedimentation rate (ESR) and C-reactive protein (CRP) may be elevated, and anemia of chronic disease may be observed. ESR and CRP do not reflect disease progression and can be normal in active TKA. At present, there are no diagnostic tests for TKA. Nevertheless, the American College of Rheumatology criteria demonstrated a sensitivity and a specificity of 90.5% and 97.8%. The presence of at least 3 of the following factors is considered suggestive of TKA: onset at age less than or equal to 40 years, claudication of an extremity, decreased brachial artery pulse, greater than 10 mmHg difference in systolic blood pressure between arms, a bruit over the subclavian arteries or the aorta, and arteriographic evidence of narrowing or occlusion of the entire aorta, its primary branches, or large arteries in the proximal upper or lower extremities [19].

### 3.3 Gastrointestinal Manifestation of TKA

The gastrointestinal manifestations of TKA mainly involve the ileum and the colon (Table 1). Additionally, splenic infarction and hepatic ischemia have been observed in TKA due to occlusion of medium and large gastrointestinal arteries [20]. In a study of 126 subjects with TKA, 16% had abdominal pain, 14% had abdominal bruits, while 4% had mesenteric ischemia. One in four patients in the study had occlusive or stenotic lesions in the celiac or superior mesenteric arteries [21]. Interestingly, another study involving 40 subjects with TKA reported elevated levels of alkaline phosphatase in three-quarters of patients, suggesting hepatic involvement [22]. Additionally, inflammatory bowel disease (IBD) has been reported to coexist with TKA. In a study with 160 subjects with TKA, 5% had IBD, and almost 70% of those patients presented with IBD 4 years before being diagnosed with to TKA [23].

**Table 1.** **Gastrointestinal manifestations of the most common etiologies of aortitis**.

Disease	Gastrointestinal manifestation	Clinical features suggesting GI involvement
Takayasu arteritis	Splenic infarction and hepatic ischemia	Abdominal pain, abdominal bruits, jaundice
	Mesenteric ischemia	
	Occlusive or stenotic lesions in the celiac or superior mesenteric arteries	
	Elevated liver enzymes	
Behçet’s disease	Mesenteric ischemia	Nausea and vomiting, dyspepsia, anorexia, melena, diarrhea, abdominal pain
	Mucosal ulcers	
	Esophageal ulcers and varices	
	Aphthous, geographic and volcano ulcers in colon	
Giant cell arteritis	Aortic aneurysm	Abdominal pain, elevated liver enzymes, nonspecific fever
	Mesenteric ischemia	
Infective aortitis	Mesenteric ischemia	High CRP and ESG
		Abnormal echocardiography
		Nausea and vomiting, dyspepsia, anorexia, melena, diarrhea, abdominal pain

### 3.4 Management

The mainstay of therapy for TKA is systemic glucocorticoids. However, long-term use of steroid is associated with significant side effects. Therefore, patients may be prescribed an immunosuppressive agent to maintain long-term remission [24]. Surgery and endovascular procedures may be indicated in cases of significant stenosis, critical ischemia, or large aneurysms.

Patients who develop new-onset arterial stenosis or major vessel inflammation (e.g., aortitis) should receive oral prednisone at a dose of 1 mg/kg per day, up to a maximum daily dose of 60 to 80 mg. This regimen should be continued for two to four weeks. High-dose intravenous steroids can be used to initiate treatment for up to three days in order to prevent impending organ failure (e.g., severe carotid or vertebral artery stenosis) [25].

As for non-steroid immunosuppressant, methotrexate and azathioprine were found to reduce the need for glucocorticoids while maintaining adequate disease control [26].

Restenosis after percutaneous angioplasty or surgical bypass is not uncommon. The rate of restenosis after open surgery reaches up to 30% at 5–20 years postop with some estimates reaching 70% [27].

## 4. Behçet’s Disease

### 4.1 Epidemiology

Another NIA is Behçet’s disease, most commonly found in the Mediterranean and Asia where 80–420 cases in 100,000 are found in Turkey alone compared to 0.12–0.64 cases per 100,000 in Western countries [28]. It commonly manifests in males in Mediterranean and Asian countries and females in Western ones [29]. The gastrointestinal manifestations of Behçet’s disease vary greatly by region with presentations in 2.8% of patients from a Turkish series, 37–43% in the US and 50–60% in Japan [29, 30].

### 4.2 Diagnosis

Behçet syndrome commonly presents with recurrent, painful mucocutaneous ulcers. Oral ulcers usually heal spontaneously within three weeks, while recurrent lesions may persist. The most specific lesions associated with Behçet syndrome are painful genital ulcers, which occur in more than three-quarters of patients [31]. Cutaneous lesions are also common and include acneiform lesions, papulo-vesiculo-pustular eruptions, pseudofolliculitis, nodules, erythema nodosum (septal panniculitis), superficial thrombophlebitis, and palpable purpura [32]. Behçet syndrome may also present with arthritis; in which case acneiform lesions are commonly found [33, 34].

Behçet syndrome affects venous and arterial vessels of all sizes, and most clinical features of Behçet syndrome are secondary to vasculitis.

### 4.3 Gastrointestinal Manifestations

Gastrointestinal manifestations of Behçet’s disease include vomiting, dyspepsia, anorexia, melena, diarrhea and abdominal pain. Behçet’s disease is also associated with intestinal perforation requiring emergency surgical intervention [29]. A distinction of the intestinal Behçet’s disease can be made between its two forms: Large-vessel vasculitis (including aortitis) causing intestinal infraction and ischemia, and mucosal ulcers from neutrophilic infiltrates mimicking IBD [35, 36]. Although the involvement of any part of the gastrointestinal tract is possible, the ileocaecal junction and terminal ileum are the most common [37]. Esophageal ulcers frequently occur in inferior esophagus, and varices have been reported in association with occlusion of the vena cava [38]. Furthermore, pyloric stenosis and ulcers present as part of the gastric manifestations of Behçet’s disease [39]. Additionally, aphthous, geographic and volcano ulcers may be found in the colon [40], and they have the highest risk of perforation in those 25 years and older [41]. In people with Behçet’s disease, 1.3–3.2% suffer from Budd-Chiari syndrome with risks increasing in young males [28]. The main determinant of survival is in this case the extent of the thrombus in the inferior vena cava. If diffuse occlusion is complete the mean survival becomes only 10 months [42].

### 4.4 Management

The goal of treatment is to suppress exacerbations and relapses in order to prevent end organ damage. Multidisciplinary management is necessary to ensure good outcomes. The European League against Rheumatism (EULAR) published guidelines on the management of Behcet Disease [43]. The recommendations can be summarized as follows:

High-dose glucocorticoids can be used for rapid suppression of inflammation during acute attacks, while regular doses can be used for gastrointestinal manifestations. Additionally, colchicine is used to prevent mucocutaneous lesion recurrence, especially if oral and genital ulcers are present. Treatment of leg ulcers, however, should involve a dermatologist and a vascular surgeon since the ulcers are usually caused by venous stasis or obliterative vasculitis. Moreover, azathioprine, thalidomide, interferon-alpha, tumour necrosis factor-alpha inhibitors or apremilast may be considered in select cases. In patients with eye involvement, an ophthalmologist should be involved.

## 5. Giant Cell Arteritis (GCA)

### 5.1 Epidemiology

GCA affects the aorta and branches of large arteries with a predilection for the vertebral and carotid branches [1]. A systematic review by Gonzalez-Gay and colleagues found that 10–25% of patients with GCA develop aortitis. Additionally, the systematic review found that GCA usually occurs in patients older than 50 years with a peak incidence in 70 and 80 years. GCA is associated with polymyalgia rheumatica, and is more common in Western countries and Caucasians [44].

### 5.2 Diagnosis

GCA has a subacute course with abrupt flareups [45]. It is often associated with constitutional symptoms including low-grade fever, fatigability, and weight loss. Headache is also a common symptom that occurs in two-thirds of patients. Headache is classically associated with scalp tenderness, but it often has no defining characteristics [46, 47]. Jaw claudication is present in about half of GCA patients. In some cases, patients notice a trismus-like symptom with restriction in the movement of the temporomandibular joint. Claudication symptoms occasionally affect the tongue during eating or with repeated swallowing [48].

The American College of Rheumatology established diagnosis criteria for GCA based on clinical and laboratory assessments in 1990. The criteria include Age at disease onset ≥50 years, New headache, Temporal artery abnormality (such as blood vessel occlusion or weakening and subsequent rupture), elevated erythrocyte sedimentation rate, and abnormal artery biopsy, i.e., non-caseating granulomatous inflammatory process along the internal elastic lamina [49]. In 2016, some authors suggested revising the criteria to a point-based system where scoring 3 or more points suggested GCA. The additional criteria included sudden onset of visual disturbances, polymyalgia rheumatica, jaw claudication, unexplained fever and/or anemia, and compatible pathology [50].

Transient monocular (and rarely binocular) visual disturbances may be an early manifestation of GCA. In transient monocular vision loss (TMVL), affected patients typically notice a sudden partial visual field loss or a transient curtain effect in the visual field of one eye. Even in the era of effective therapies, the incidence of permanent partial or complete loss of vision in one or both eyes due to GCA, as described by several centers, is between 15 and 20 percent of patients [51, 52, 53, 54, 55, 56]. Permanent vision loss may be preceded by single or multiple episodes of transient vision loss, but it may also occur with devastating rapidity. Once vision loss has occurred, it is rarely reversible [57]. In addition, it is estimated that 25 to 50 percent of untreated patients will experience further loss of vision in the unaffected eye within one week. Nevertheless, prompt initiation of appropriate steroid treatment virtually eliminates the risk of subsequent vision loss. If vision loss is already present, such treatment significantly reduces the risk of further deterioration but does not improve the existing vision loss [58].

Large vessel (LV) involvement in GCA causes aneurysms and dissections especially in the thoracic aorta. Stenosis, occlusion, and ectasia of large arteries have also been described [59]. Authors studied 40 patients with confirmed GCA using computed tomographic (CT) angiography and found evidence of large-vessel vasculitis (including the aorta and/or its tributaries arteritis in two-thirds of patients. Authors defined aortitis as circumferential aortic wall thickness ≥2 mm with or without contrast enhancement of the vessel wall observed in zones without adjacent atheroma. The aortic tributaries including the brachiocephalic trunk, carotid, subclavian, axillary, splanchnic (coeliac and mesenteric), renal, iliac and femoral arteries were also evaluated. Radiological findings considered included circumferential wall thickness, contrast enhancement of the artery wall, arterial diameter and the presence of stenoses. Arteritis was considered to be present when the thickness of the artery wall was >1 mm. Sixty-five percent of those patients in the study had aortitis, 47 percent had brachiocephalic trunk involvement, 42% subclavian arteries and 30% had femoral arteries vasculitis. [60]. Clinical recognition of aortic aneurysms/dilatation has been described in 10 to 20 percent of cases [61, 62, 63, 64]. The thoracic aorta, especially the ascending aorta, is affected more often than the abdominal aorta. Nevertheless, major complications such as aortic dissection and rupture occur less frequently [61, 62].

### 5.3 Gastrointestinal Manifestations

In patients with GCA, abdominal pain can result from abdominal aortic dissection or aneurysm. A cohort study from a clinic in Minnesota followed 96 patients who developed GCA between 1950 and 1985. Authors reported aortic artery aneurysms in 11.5% of patients. Most of those patients developed aortic aneurysms after a median of 6 years from diagnosis [65]. Thus, patients diagnosed with GCA should have regular screening for aortic aneurysms at the time of diagnosis and throughout follow-up [66].

GCA has also been shown to affect the liver. Twelve of 56 patients with GCA who were followed in Jerusalem had elevated liver enzymes including alkaline phosphatase and transaminase levels [67]. These elevated levels could be a result from bile duct epithelial cells being injured due to neighboring arteritis [68].

GCA rarely affects the mesenteric vessels. A literature review in 2008 found 12 cases of GCA with mesenteric involvement [69]. Fifty percent of these cases had predominating abdominal symptoms with a less common occurrence of cranial symptoms. Some cases of large bowel infarction infraction of the large bowel were described. In such cases, patients usually present with in the literature and present with nonspecific fever, acute abdomen or abdominal pain. Some extremely rare occurrences are that of granulomatous inflammation of the liver and the portal tract hepatic arteritis that can induce gastrointestinal symptoms and fever before the cranial symptoms that are suggestive of GCA [70, 71].

### 5.4 Management

Glucocorticoids are the treatment of choice for GCA. In patients with a positive biopsy, high-dose systemic glucocorticoids are the mainstay of therapy and should be instituted promptly once the diagnosis of giant cell arteritis (GCA) is strongly suspected, especially in patients with recent or threatened visual loss. A temporal artery biopsy or other diagnostic procedure should be obtained as soon as possible, but treatment should not be withheld while awaiting the performance or results. In cases where the clinical scenario for GCA is compelling but the diagnostic workup is negative, the diagnosis of GCA may be arrived at on clinical grounds.

GCA is treated with daily glucocorticoids [72]. Adjuvant treatment with tocilizumab or methotrexate may be used to avoid steroid side effects [73]. These options are indicated in patients with significant co-morbidities, in those with significant corticosteroid side effects, and when a relapse necessitates prolonged immunosuppression.

In case of severe gastrointestinal manifestations, an immunosuppressant is usually used with a steroid. Surgery and endovascular procedures are used in an as-needed basis. Surgical treatment should be considered in patients who develop an aortic aneurysm, ideally in the dormant phase of the disease. Owing to the morbidity risk associated with surgical repair of GCA-related aneurysms, we recommend performing it only in specialized, experienced tertiary care centers. Endovascular repair has also been reported for aortic aneurysms. Endovascular repair can be considered for particularly ill patients and provides them with superior short-term outcomes compared to those undergoing open surgery [74].

It is noteworthy that patients suffering from GCA tend to be older than those suffering from Takayasu arteritis, which is why the morbidity and mortality of GCA is higher [75]. Adjunctive methotrexate could reduce relapse as well as reliance on steroids [76]. The use of tocilizumab has also been studied in clinical trials; It was found that 85% of patients with GCA experience sustained remission within one year, and 80% of patients are able to discontinue glucocorticoids [77].

## 6. Infective Aortitis (IA)

### 6.1 Epidemiology

The aorta is normally resistant to infection. Risk factors for infective aortitis include atherosclerosis, syphilis, cystic medial necrosis, and aortic prosthesis. IA is more frequent in med and elderly patients. It usually presents with aneurysmal disease or infective endocarditis [78]. Infectious aortitis is an uncommon finding, representing only 2.6% of all abdominal aortic aneurysm.

Infection may follow septic embolization of the aorta (“embolomycotic”), hematogenous seeding (“microbial aortitis or infected aneurysm”), or spread from a contiguous focus of infection. The mortality associated with infectious aortitis usually ranges from 21% to 44%, higher if managed with antibiotics alone. Increased mortality is associated with uncontrolled infection or sepsis, infection with more virulent microorganisms, suprarenal extension of the aneurysm, and perhaps aneurysm rupture, whereas 30-day mortality may be decreased in patients who are revascularized using cryopreserved arterial homografts [79].

### 6.2 Diagnosis

Patients usually present with a fever, back, chest, or abdominal pain, pulsatile abdominal mass, leukocytosis, and a positive blood culture. Diagnosing infectious aortitis requires a high index of suspicion since symptoms and signs are nonspecific.

Blood cultures can help identify bacterial causes of aortitis. However, signs on CT scan can help guide a diagnosis. CT scan is rapidly indicated for patients with suspected abdominal aortic aneurysm, which frequently accompanies aortitis. Features that can be identified periaortic soft tissue or fluid accumulation, aneurysmal dilatation, and vertebral body osteomyelitis. Other diagnostic options include magnetic resonance imaging and nuclear medicine scintigraphy. Additionally, transesophageal echocardiography may provide insight into the thoracic aorta.

### 6.3 Gastrointestinal Manifestations

Diagnosis often occurs after suspicion from the patient’s symptoms and history supported by peculiarly high c-reactive protein and erythrocyte sedimentation rate. The presentation is usually non-specific and for this reason a high index of suspicion must be maintained. Symptoms include pyrexia of unknown origin, abdominal and/or back pain, palpable pulsatile abdominal mass, and signs of rupture abdominal aortic aneurysm rupture. Hemorrhage into the gastrointestinal tract, which manifests in hematemesis, coffee ground vomitus, and/or melena occurs in patients with bowel erosion or an aorto-enteric fistula. Imaging such as echocardiography also guides the diagnosis [2]. Salmonella spp. are the most common bacteria causing abdominal aortitis. However, two-thirds of cases of aortitis in developing countries are due to Mycobacterium tuberculosis.

### 6.4 Management

IA is managed by adequate antibiotic therapy depending on the infectious agent in question. Broad-spectrum antibiotics may be used while waiting for blood culture results [80]. Hospitalization with extensive workup is indicated for any adult, especially over the age of 50 years, who presents with fever, chest or abdominal pain, and positive blood cultures in which the diagnosis of infectious aortitis is suspected [81]. Any patient with fever associated with a palpable aneurysm should also be hospitalized, because rapid evaluation and diagnosis are required to avoid aneurysm rupture. Aneurysms due to gram-negative infections are associated with a greater tendency toward early rupture than those associated with gram-positive infections (84% vs 10%) [82].

If surgical intervention is immediately planned, antibiotics should be initiated after intraoperative cultures are obtained. Because gram-negative bacteria, like Salmonella species, and gram-positive organisms, like *S. aureus*, are the most commonly isolated bacterial pathogens, initial antibiotic selection should be active against these bacteria. The duration of antimicrobial therapy is usually 6 to 12 weeks, possibly 1 year or indefinitely in the immunocompromised patient; however, controlled trials are lacking [83, 84]. Rifampin impregnated grafts have been used successfully in a limited number of patients [85]. However, it must be emphasized that treatment of infectious aortitis requires a combined medical and surgical approach.

The goals of surgical therapy are removal of infected tissue, often including an aneurysm resection, and restoration of distal arterial flow [86]. This should be followed by long-term systemic antibiotic therapy. Overall, the surgical mortality rates range from 40% to 45%, much of which is influenced by the presence of vessel rupture prior to surgery, whether the infection involves an existing prosthetic aortic graft, and the suprarenal extent of the aneurysm.

## 7. Rare Causes

According to the International Chapel Hill Consensus Conference on the Nomenclature of Systemic Vasculitides [1], this condition can be classified into large, medium, and small vasculitis. However, small- and medium-vessel vasculitis can also affect the aorta, although this is rare. For instance, Veraldi and colleagues reported the case of a 46-year-old man who was admitted for investigation of an abdominal aortic aneurysm with the presence of solid fibrous inflammatory tissue surrounding the aortic wall. Authors suspected infective or autoimmune etiology. They performed a laparotomy during which they noted extensive solid fibrous tissue surrounding the aorta was found without any cleavage planes between anatomical structures. For this reason, they performed aneurysmectomy, in-situ revascularization with an arterial homograft, and obtained periaortic specimens for histopathologic examination. The histological specimens confirmed the presence of vasculitis lesions, associated with eosinophilic and plasma cellular infiltration. The patient was diagnosed with Anti-neutrophil cytoplasmic antibody (ANCA) vasculitis complicated by symptomatic infrarenal aortic aneurysm was concluded. He responded well to therapy with a glucocorticoid in addition to methotrexate and was discharged 3 weeks after surgery [87].

## 8. Conclusions

Aortitis may present with gastrointestinal manifestations. While this is rare, it could quickly become life-threatening and physicians must therefore maintain a high index of suspicion. A multidisciplinary protocol must be put in place to improve patient prognosis.
